# Oncoral Follow-Up for Outpatients Treated with Oral Anticancer Drugs Assessed by Relative Dose Intensity

**DOI:** 10.3390/ph18040565

**Published:** 2025-04-13

**Authors:** Virginie Larbre, Florence Ranchon, Delphine Maucort-Boulch, Elsa Coz, Chloé Herledan, Anne-Gaëlle Caffin, Amandine Baudouin, Magali Maire, Nicolas Romain-Scelle, Charles-Hervé Vacheron, Lionel Karlin, Gilles Salles, Hervé Ghesquières, Catherine Rioufol

**Affiliations:** 1 Clinical Oncology Pharmacy Unit, Lyon Sud Hospital, Hospices Civils de Lyon, 69495 Pierre Bénite, France; virginielarbre@yahoo.fr (V.L.); florence.ranchon@chu-lyon.fr (F.R.); chloe.herledan@chu-lyon.fr (C.H.); anne-gaelle.caffin@chu-lyon.fr (A.-G.C.); amandine.baudouin@chu-lyon.fr (A.B.); magali.maire@chu-lyon.fr (M.M.); 2 EA 3738—CICLY, Claude Bernard Lyon 1 University, 69921 Oullins, France; 3 Pôle Santé Publique, Service de Biostatistique et Bioinformatique, Hospices Civils de Lyon, 69002 Lyon, France; delphine.maucort-boulch@chu-lyon.fr (D.M.-B.); elsa.coz@chu-lyon.fr (E.C.); charles-herve.vacheron@chu-lyon.fr (C.-H.V.); 4 UMR CNRS 5558, LBBE Laboratoire de Biométrie et Biologie Évolutive, 69622 Villeurbanne, France; nicolas.romain-scelle@univ-lyon1.fr; 5 Department of Hematology, Lyon Sud Hospital, Hospices Civils de Lyon, 69495 Pierre Bénite, France; lionel.karlin@chu-lyon.fr (L.K.); herve.ghesquieres@chu-lyon.fr (H.G.); 6 Lymphoma Service, Department of Medicine, Memorial Sloan Kettering Cancer Center, New York, NY 10065, USA; gilles.salles@mskcc.org; 7 CIRI Centre International de Recherche en Infectiologie, INSERM U1111, Claude Bernard Lyon 1 University, 69921 Oullins, France

**Keywords:** drug safety, risk management, care coordination, oral anticancer drugs

## Abstract

**Objectives**: The multidisciplinary city-hospital Oncoral follow-up of cancer outpatients has been set up to ensure the safety of oral anticancer drugs (OADs). The aim of this study was to assess Oncoral by Relative Dose Intensity (RDI) in patients with hematological malignancies treated with ibrutinib as a model. **Methods**: The study included all outpatients treated with ibrutinib and followed in Oncoral between January 2016 and June 2020. Patients benefited from interviews leading to pharmacist and nurse interventions (PNI) on drug-related problems as adverse events (AE), drug–drug interactions (DDI), and drug intake. **Results**: In total, 83 patients were enrolled. At least one PNI was performed for 86.7%, focusing on drug intake and DDIs (54.5%), the management of AEs (27.0%), and community–hospital coordination (18.5%). Major DDIs with ibrutinib were found in 10 patients, with at least one moderate interaction in 28%. Grade 3–4 AEs mainly concerned cytopenia and infection. Adherence tended to decrease after the first 6 months. At 6 months, the mean RDI was 93.7 ± 11.3%; RDI reductions occurred in 43% patients. RDI was lower in patients who discontinued treatment before day 90 and worsened over time in patients still being treated at month 6 (Friedman’s test, *p* < 0.01). Age and gender were predictors of early treatment termination (OR 1.10 [1.03; 1.19] and 6.44 [1.65; 37.21]). The estimates of 30-month OS and PFS were 73.8% (95% CI [64.7%; 84.2%]) and 61.8% (95% CI [51.8%; 73.7%]). **Conclusions**: The Oncoral follow-up is a secure, coordinated pathway assessed by RDI. Multidisciplinary follow-up should be the gold-standard for outpatients receiving OADs.

## 1. Introduction

Oral anticancer drugs (OADs) have drastically changed the treatment of cancer. However, drug-related problems (DRPs) such as non-adherence, side effects, and drug–drug interactions (DDIs) compromise the quality of care [1]. Follow-up programs involving hospital–community coordination have been consequently set up, leading to positive impacts on drug safety and risk management, with reductions in DRPs and unplanned admissions. Such benefits are improved by monitoring patient-reported outcomes (PROs), allowing for early management [2,3,4,5,6,7,8,9,10,11,12,13,14]. Two randomized comparative studies demonstrated significant reductions in severe AEs and hospital stay versus standard of care. The multicenter Ambora study assessing follow-up by pharmacists and pharmacologists reported a reduction in DRPs, including errors and DDIs, and significant improvement in adherence [15]. However, except for these studies, the impacts of the multidisciplinary programs set up to follow up patients treated by OADs remain poorly assessed due to the lack of a gold standard. Subsequently, the Capri study assessing nurse-led telemonitoring showed a significant increase in Relative Dose Intensity (RDI) [16]. The authors reported that RDI should exceed 85% to improve outcomes, in accordance with previous studies with intravenous chemotherapy or OAD [17,18,19,20,21,22,23,24,25].

Studies in patients treated for adjuvant and metastatic breast cancer were the first to demonstrate a relationship between RDI and survival [26,27]. This association has been confirmed by subsequent prospective studies in patients with hematological malignancies such as chronic myeloid leukemia [28] and lymphoma [29]. Other studies on different types of cancer show that Relative Dose Intensity correlates with therapeutic efficacy, and that overall survival and/or progression-free survival are reduced for relative dose intensities below 0.80–0.90 [30,31,32].

In patients treated with OAD, an RDI of greater than 70% was associated with better disease control, longer time to treatment failure and longer OS [33,34]. The Resonate III study reported a higher threshold of RDI ≥ 80% for improved progression-free survival (PFS) in patients with chronic lymphocytic leukemia (CLL) and lymphoma treated with ibrutinib [35].

Oncoral monitoring is based on multidisciplinary community–hospital interventions by hospital oncologists, pharmacists and nurses coordinating with primary care providers. Regular face-to-face interviews with the patients focus on drug intake and adherence, the prevention and management of symptoms, side effects, and DDI. A teleconsultation platform is available daily for any questions. In addition, pharmacist and nurse interventions (PNIs) improve the community–hospital coordination of patient care. All previous studies support the Oncoral choice of RDI as the primary endpoint. In addition, despite the growing number of multidisciplinary care pathways for patients treated with oral cancer drugs, none have been evaluated using an objective and robust criterion such as RDI. This study is the first to assess the Oncoral follow-up of outpatients treated with OADs using Relative Dose Intensity (RDI). The homogeneous population of patients treated with ibrutinib for CLL and other lymphoid malignancies was selected to describe the model.

## 2. Results

### 2.1. Patient Characteristics

In total, 83 eligible patients were enrolled, with a median interval of 6.8 years from diagnosis to ibrutinib initiation (Table 1).

### 2.2. Medication and Complementary and Alternative Medicines (CAM)

At the initiation of ibrutinib, polypharmacy was reported in 36.9% of patients (n = 24), including 5 patients (20.8%) with excessive polypharmacy. The mean number of chronic drugs per patient was 4.2 ± 3.2 (1–13), mostly cardiovascular (69.2%) or neurologic (43.1%). Further, 40.9% of patients were taking ≥1 psychotropic drugs during follow-up; 28.9% were treated with anti-thrombotic or anti-hemorrhagic agents, including 14 taking curative or prophylactic antiplatelets. In total, 62 patients (74.4%) used at least one CAM during follow-up, with a mean 4.1 ± 5 CAMs per patient, to manage digestive disorders, pain, inflammation, stress or anxiety, or to improve their general health condition and cancer therapy. Half of CAMs were biologically-based therapies including natural products, particularly herbal medicine, as used by half the patients, followed by complementary medical systems (27%) such as acupuncture, homeopathy or naturopathy.

### 2.3. Pharmacist and Nurse Interventions

At least 1 PNI was performed for 86.7% of patients (n = 72), for a total of 339 PNIs and a mean of 4.7 ± 4.5 per patient. Further, 270 PNIs (79.6%) were associated with ibrutinib. The interventions focused on drug or CAM intake and DDIs (54.5%), the management of AEs (27.0%), and community–hospital coordination (18.5%). PNIs were first related to approved requests by patients or caregivers for drugs or CAMs, whether prescribed or not (n = 84, 31.1%), resulting in 60 approvals (71.4%) (antibiotics, proton pomp inhibitors, etc.) and 21 (25.0%) interruptions of CAMs (turmeric, desmodium, etc.). Having more than five PNIs was not predictive of ETT on univariate analysis (*p* = 0.587).

PNIs concerning drug use and DDIs were most often prompted by an increased risk of bleeding (Table 2). Major DDIs involving ibrutinib were found in 10 patients; 5 DDIs increased the risk of bleeding by interfering with platelet function or coagulation and 5 potentialized the risk of impaired ibrutinib metabolism by concomitant inhibitors or inducers of CYP450 3A4. At least one moderate interaction with ibrutinib was identified in 28% of patients (n = 23). PNIs were performed in cases of major and moderate interaction, depending on the patient, clinical situation and pharmacological relevance. The DDIs leading to PNIs are presented in Table 2.

**Figure 1:**
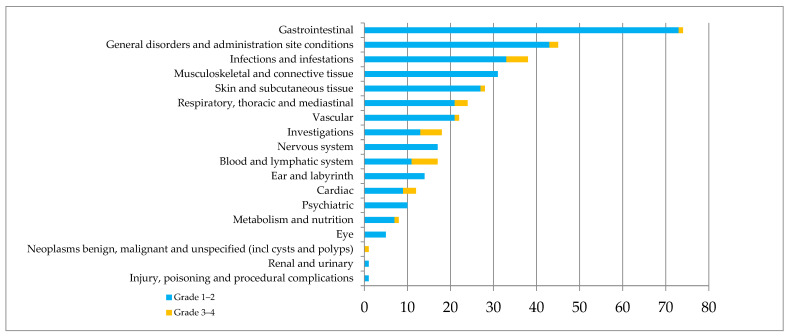
Safety of Ibrutinib during the first 6 months of treatment according to CTCAE classification.

Secondly, PNIs concerned the management of AEs, leading to 43 recommendations (58.9%) to patients or caregivers (e.g., management of pain, cramps, digestive disorders, etc.), 17 requests (23.3%) for a hematologist’s opinion (e.g., bleeding, aphtha, etc.), and 10 referrals to specialist consultation (13.7%). Almost all patients experienced at least one AE during the first 6 months of treatment (Figure 1).

Half of patients experienced symptomatic treatment for at least one AE during the first 6 months. Nearly three-quarters of reported AEs occurred during the first 3 months (n = 272), and 50% of patients experienced between 1 and 5 AEs. The median number of AEs was 2 in the period 0–90 days, compared to 0.5 in the period 91–180 days. Grade 3–4 AEs (n = 31, 8.4%) mainly concerned cytopenia and infection, with 20 and 11 serious AEs reported for the periods 0–90 days and 91–180 days, respectively, leading to definitive treatment withdrawal in five patients. The prescription of ibrutinib was modified for 40 patients (48.2%) due to AEs leading to temporary withdrawal (n = 24, 42.9%), dose reduction (n = 16, 28.6%), temporary withdrawal followed by resumption at a lower dose (n = 6, 10.7%), or definitive withdrawal (n = 10, 17.9%). In 15 cases, AEs led to a modified concomitant treatment. In total, 31 patients (37.4%) developed cardiovascular AEs (n = 34, 9%); 9 (26.4%) led to definitive or temporary withdrawal of treatment or dose reduction.

Regarding specific AEs, 31 patients (37.4%) developed cardiovascular AEs (n = 34, 9%)—hematoma (n = 14, 41.2%), tachycardia (n = 5, 14.7%), arrhythmia (n = 4, 11.8%), thrombosis (n = 3, 8.8%), heart failure (n = 3, 8.8%), bleeding (n = 2, 5.9%), bruising (n = 2, 5.9%), hyper- or hypotension (n = 2, 5.9%), or stroke (n = 1, 2.9%). Most were mild (grade 1) (n = 25, 74%). Nine (26.4%) cardiovascular AEs led to the permanent discontinuation of treatment (n = 4, 11.8%), temporary withdrawal (n = 3, 8.8%), dose reduction (n = 1, 2.9%), or temporary withdrawal followed by dose reduction (n = 1, 2.9%).

Adherence was optimal or moderate during the first 6 months, then tended to decrease with time, from 74% of optimal adherence at month 1 to 58% at month 12. Around 25% of patients declared moderate adherence. Median MPR at month 6, assessed in 69 patients, was 100 ± 0.16%. In addition, PNIs contributed to the assessment of compliance through consultations. Respectively, 39, 42 and 26 adherence questionnaires were completed at days 0–60, 61–150 and 151–210 in patients with ETT at the 180-day landmark.

### 2.4. Relative Dose Intensity

Mean RDI was 95.3 ± 11.3% at month 1, 94.6 ± 10.5% at month 3, 93.7 ± 11.3% at month 6, 93.9 ± 11.0% at month 9, and 93.2 ± 12.7% at month 12. At 6 months, the median RDI was 100%, with RDI < 85% in 10 patients out of 64 (15.6%). The reasons for these increases in RDI were an increase in prescribed dose (n = 5) and the assurance of safety after monitoring for concomitant therapy. The reasons for RDI reductions were AEs (n = 30), leading to 10 dose reductions; 13 temporary withdrawals (3 plus dose reduction) and 7 cessations of ibrutinib; surgery (n = 8) systematically leading to temporary withdrawal; and stable disease (n = 1) leading to cessation of ibrutinib. RDI reductions at month 6 occurred in 43% patients (n = 36) due to initial or subsequent dose reductions according to clinical status, with the temporary or permanent discontinuation of ibrutinib. Patient characteristics according to RDI at day 180 are presented in Table 3.

**Figure 2:**
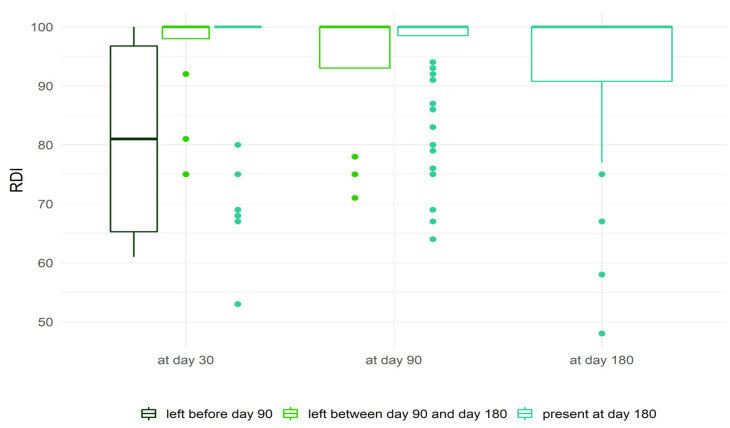
Relative Dose Intensity (RDI) at different follow-up times in patients stopping ibrutinib before day 90 and between day 90 and day 180, and those still treated at day 180.

The boxplot in Figure 2 displays RDI distribution at days 30, 90 and 180 for patients discontinuing treatment before day 90, and between days 90 and 180 for patients still treated at day 180. In patients who discontinued treatment before day 90, RDI was lower than in the other two groups. In patients still treated at month 6, RDI significantly worsened over time (Friedman’s test, *p* < 0.01). This graph illustrates the immortal-time bias, as patients with lower RDI are shown to be more at risk of stopping treatment.

**Figure 3:**
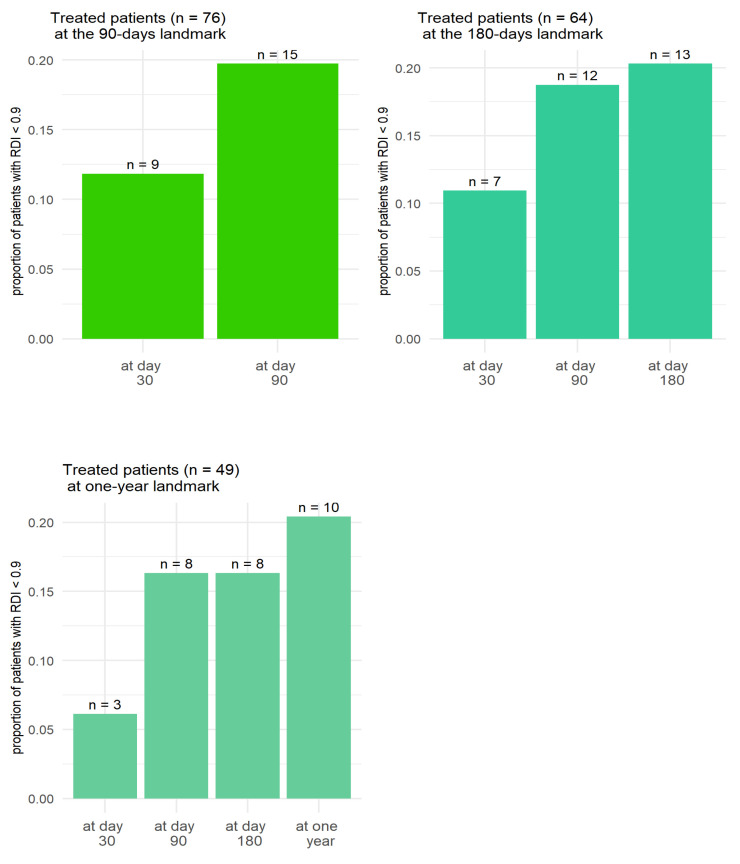
Proportion of patients with RDI < 90% at the 90-day, 180-day and one-year landmarks.

For the evaluable patients, RDI < 90% was more frequent at month 3 than before. RDI was more often degraded between months 1 and 3 than between months 3 and 6 (Figure 3).

RDI at month 12 was not assessed in 34 patients (41%) because ibrutinib was previously definitely stopped mostly due to disease progression (41.2%, n = 14), AEs (29.4%, n = 10), or stable disease (5.9%, n = 2). Ibrutinib interruptions occurred during the first month, between 1 and 3 months, between 3 and 6 months, between 6 and 9 months, and between 9 and 12 months, respectively, for eight, five, eight, eight, and seven patients; the average durations of interruption were, respectively, 0.8 ± 2.5, 1.4 ± 4.6, 3.3 ± 11.4, 2.9 ± 12.9 and 3.6 ± 9.8 days.

Age was significantly linked with RDI values. The mean age of patients who discontinued treatment after 6 months was 77.38 ± 8.83 years, compared with 71.17 ± 9.90 years for those who did not (*p* = 0.009). Male gender was a risk factor for the discontinuation of oral cancer therapy before 6 months (*p* = 0.027).

### 2.5. Corrected Relative Dose Intensity

The mean RDIc values at 6 and 12 months were, respectively, 95.8 ± 9.9% and 95.3 ± 11.8%. During the first 6 months, doses were adjusted according to drug approval for 10 patients (15.6%): 6 due to surgery, 2 due to AEs (grade 3 neutropenia with infection and grade 4 hematological toxicity), 1 due to surgery plus AEs and due 1 to clinical recommendations. The mean RDIc at 6 months for these patients was 97.9%.

### 2.6. Factors for Early Treatment Termination

At month 6, 19 patients had stopped their treatment. Table 4 shows the results of univariate and multivariate analyses of ETT risk according to baseline characteristics.

Age and gender were strong predictors of ETT (OR 1.10 [1.03; 1.19] and 6.44 [1.65; 37.21]), as were hematological malignancy and CCI. AE grade was not predictive of ETT on univariate analysis (*p* = 0.163).

### 2.7. Progression-Free and Overall Survival

**Figure 4:**
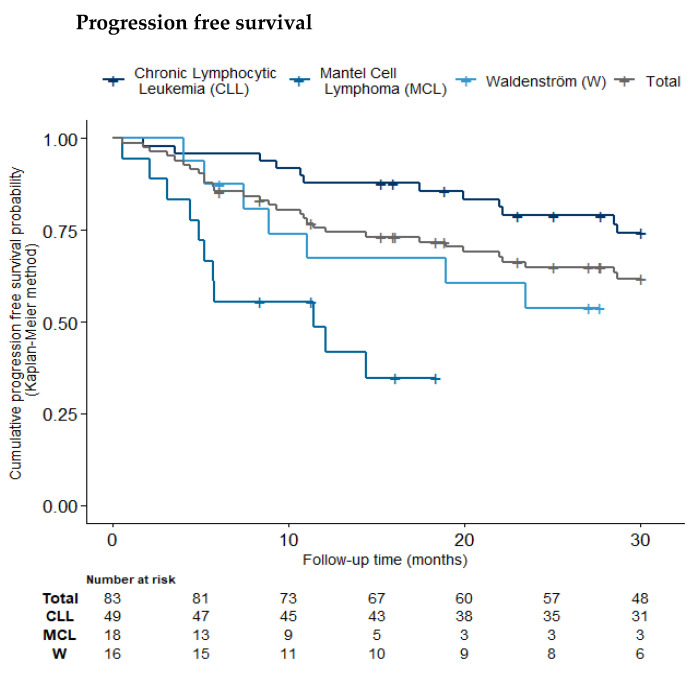

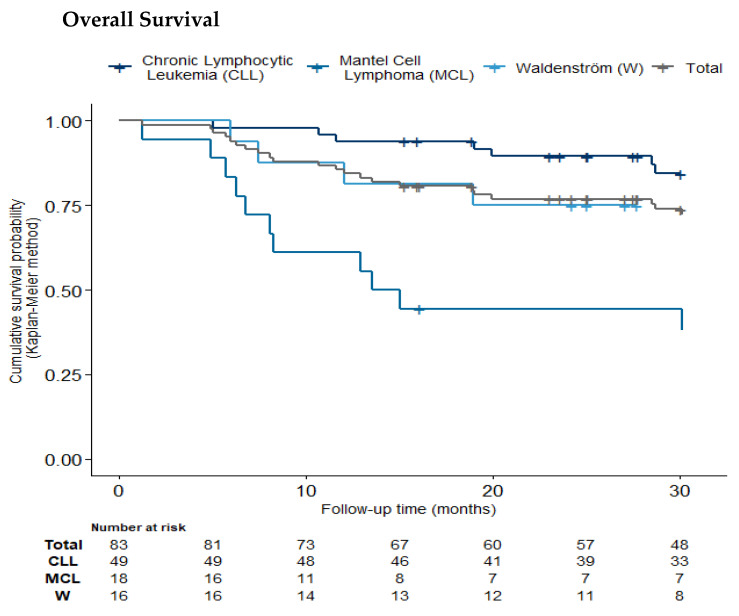
Overall survival and progression-free survival at 30 months displayed by type of cancer (CLL, MCL, WD).

PFS and OS were estimated up to 30 months (Figure 4). Follow-up data for all-cause mortality were available for 83.1% of patients at 30 months. The Kaplan–Meier estimate of 30-month overall survival probability was 73.8% (95% CI [64.7%; 84.2%]). The estimate for progression-free survival was 61.8% (95% CI [51.8%; 73.7%]).

## 3. Discussion

This study highlights the RDI-assessed Oncoral follow-up as a secure and coordinated pathway in outpatients treated by OADs. The strong points of this follow-up include the association of oncologists with pharmacists and nurses, and improvements in the city–hospital coordination, securing the care pathway in clinical practice, given that most programs rely simply on hospital nurses or pharmacists [24,36,37].

Oncoral includes polymedicated patients (37%) taking drugs at risk of interaction with the OAD [38,39,40,41]. Despite the risks of DRPs, anticancer treatment should be maintained. Drug reconciliation and patient education are therefore essential to secure drug intake, and to proposing alternative treatments.

There is no gold standard for evaluating the follow-up of outpatients treated with oral anticancer drugs, and adherence remains a subjective criterion. RDI gives an overview of drug intake, including dose reductions, and temporary and definitive withdrawals. The relationship between RDI and survival was consequently assessed [17,35,42,43,44]. With mean RDIs of, respectively, 93.7% and 93.2% at 6 and 12 months, the Oncoral monitoring allows optimal and stable drug exposure. These real-life results are similar to those obtained in clinical trials through the strict monitoring of patients, reporting an overall mean RDI of 95% or median RDI of 98% [35,45]. The RDI at 6 months of Oncoral monitoring was similar to the 93.4% RDI reported in the interventional arm in the Capri study, and also greater than the 85% threshold identified by the authors as ensuring optimal treatment [16]. However, the present study only concerns patients with hematological malignancies, and in order to be comparable with the CAPRI population, should be applied to patients treated for any cancer (hematological malignancies and solid tumors).

Another study reported that above the 80% threshold, RDI impacts PFS in CLL patients treated with ibrutinib [35]. These findings suggest the security provided by Oncoral. In addition, this real-life follow-up provides complementary information to clinical trials, suggesting that a low RDI in the first 3 months can predict the early discontinuation of OAD; this is crucial for therapeutic decision-making, and needs confirmation in order to be applied in everyday practice. Predictive factors for RDI reduction comprise hematological lymphoid malignancy subtypes, gender, age and CCI, which complement previous studies, which identified obesity or high body mass index [46]. In addition, this study provides survival data in real time, with PFS and OS similar to those of a comparable population [5]. The correlation between ibrutinib RDI and survival needs to be further assessed.

In addition, the present study is the first to illustrate the immortal-time bias, as patients with lower RDI are more at risk of stopping treatment. This is a major result which can be further investigated to identify factors predictive of discontinuation and to optimize the medical decision process.

This study is the first to assess RDIc, considering modifications according to the SPC. At months 6 and 12, the RDIc was 95.8% and 95.3%, respectively, confirming that the hematologists followed recommendations, especially for treatment withdrawal before surgery. Some modifications were made empirically as a precaution, e.g., temporary treatment withdrawal in grade 2 hematoma. Optimal RDI is crucial to the outcome, and the physician’s clinical opinion must be considered for each patient, leading to personalized treatment.

The large proportion of patients requiring PNIs (86.7%) and their mean number per patient (4.7 ±4.5) argue for the usefulness of Oncoral, with multidisciplinary interventions on drug administration schedules and interactions for more than half of the PNIs (54.5%), prevention and management of AEs for nearly a third (27%), and community–hospital coordination for almost 20%. The interactions of OAD with phytotherapy or food should not be underestimated [5,47,48]. In this study, three out of four patients used CAM, with a mean 4.1 products per patient, half of which involved phytotherapy, potentially modifying the efficacy or toxicity of OAD. The intervention of the pharmacist is all the more important as patients rarely report the use of phytotherapy to the physician [49,50,51]. In addition, Oncoral controls DRPs, in agreement with the multicenter Ambora study, in which a mean of 1.7 DRPs per patient were detected in the first 12 weeks of treatment [15]. In addition to DDI, it would have been interesting to compare RDIs in the present study with those in the population of the Ambora study, but this was not possible as the Ambora study did not collect RDIs.

Likewise, 75% of AEs were detected during the first 3 months of treatment, with twice as many grade 3–4 severe AEs as in the following 3 months, confirming the need for secure follow-up from the outset of treatment [52,53]. Even so, other DRPs were detected later, notably by PROs and community–hospital coordination. Patient and community pharmacy requests about drug interactions in the case of a change in the prescription provide important inputs. Thus, DRP follow-up must be reinforced during the first months and maintained to ensure continued security. The pattern of adherence, with 74% showing good adherence after the first month and 58% at 12 months, is a further argument for sustaining the pathway continuously. Moreover, in the absence of a gold standard for measuring adherence, and given that electronic pill boxes are poorly suited to real-life studies, the simultaneous use of two subjective methods allows us to increase the reliability of the results.

In the present study, PROs were collected by phone or teleconsultation between hospital visits. However, it is crucial to incorporate telemonitoring, enabling greater outcomes with improved quality of life [7,47,54,55,56]. Other study limitations include the monocentric design and lack of a control group; however, real-world data are scarce, complementing clinical trials. However, the survival results for real-life cancer patients differ from those reported in clinical trials [57]. Thus, despite the monocentric design, one of the strengths of this study is that it provides real-life data that are known to illustrate the conditions under which drugs are used in routine care, with more heterogeneous populations, more indications, and more patient profiles that are more complex to treat because of comorbidities and polypharmacy, compared to clinical trials. In addition, real-world studies can produce results more quickly than clinical trials [58]. In recent years, there has been a consensus on the usefulness of high-quality data from large cohorts used to strengthen and improve research methods and practice [59]. Health decision-makers increasingly seek real-world evidence for market authorization and pricing, as well as clinicians to improve outcomes. RDI and adherence were calculated separately; unfortunately, no method today provides combined criteria. Such follow-up benefits cancer patients, but is time-consuming and requires human resources [60], which will need to be assessed in the future. The impact of psychosocial vulnerability on RDI has to be further investigated, as other social factors may also impact follow-up [48].

## 4. Materials and Methods

### 4.1. Study Design and Population

This prospective study was conducted in the Hematology Department of Lyon University Hospital (France) from January 2016 to June 2020. Eligible patients were adult outpatients treated with ibrutinib for CLL, mantle cell lymphoma (MCL) or Waldenström’s macroglobulinemia (WM), followed in the Oncoral program.

Oncoral (oncological care for outpatients with oral anticancer drugs) is a city-hospital multidisciplinary program based on educational interviews and follow-up performed by the oncologist or hematologist, the clinical pharmacist and the nurse, with the involvement of the general practitioner and the community pharmacist. Interviews address understanding the medication plan, as well as the management and prevention of side effects and drug interactions, including other prescribed drugs, self-medication and Complementary and Alternative Medicines (CAM). At the initiation of OAD, medication reconciliation was performed. Patients were asked about all the drugs and CAM used. The collected data were completed using different sources of information, such as medical records and available patient prescriptions. Polypharmacy was defined as the intake of five or more medications daily, and excessive polypharmacy as the intake of ten or more medications daily. A medication review was performed to detect drug interactions using the following databases: Drugs.com^®^ for drugs, and Hedrine^®^ and/or Memorial Sloan Kettering Cancer Center (MSKCC) database for plants. Interactions were classified according to Drugs.com^®^ with three different risk levels—major, moderate and minor. If DDIs were detected and appeared clinically relevant, the pharmacist informed the prescriber and an appropriate decision was taken. Therapeutic information focusing on the medication plan, AEs and DDI was shared via secure messaging between the hospital team, the community pharmacist and the general practitioner.

The follow-up based on monthly interviews was carried out for the entire duration of the OAA treatment [61]. Interviews last between 20 min and 1 h. The pharmacists and nurses involved in oncology follow-up had at least 1 year of experience in cancer care, either in an oncology pharmacy unit (pharmacists) or in an oncology ward (nurses). All had completed a 40 h training course on therapeutic education (Hospices Civils de Lyon).

Data were collected over the 12 months following treatment initiation. Eligible patients had to have received ibrutinib for at least 6 months, as estimated by the hematologist. Exclusion criteria comprised being a non-French speaker, needing professional caregivers to take drugs, and living in an institution. By agreement with the investigators and sponsors, patients enrolled in a clinical trial were not eligible for follow-up with Oncoral, and therefore not eligible for this study, in order to avoid bias in the methodology of clinical trials. All participants provided written informed consent to the processing of their personal data in accordance with the Declaration of Helsinki. The study was registered in the National Data Protection Commission register authorized for Hospices Civils de Lyon (number 15-122).

The data collected were completed by use of patient interviews, concerning medical records and prescriptions. Polypharmacy was defined as the intake of 5 or more drugs daily, and excessive polypharmacy as the intake of 10 or more.

Safety outcomes were assessed by the occurrence of AEs and serious AEs detected during the follow-up and regular consultations. Serious AEs are defined as any medical event that results in death, is life threatening, requires inpatient hospitalization or causes the prolongation of existing hospitalization, results in persistent or significant disability, could cause a congenital anomaly, or requires intervention to prevent permanent impairment or damage. The Common Terminology Criteria for Adverse Events version 4.0 was used, and AEs were graded from 1 to 5 [62]. Focus was placed on patients with a history of cardiovascular disease, as ibrutinib should be used with caution in these cases.

A medication review was performed to detect DDIs, using the Drugs.com^®^ database for drugs, and the Hedrine^®^ and/or Memorial Sloan Kettering Cancer Center (MSKCC) databases for plants. Interactions were classified according to Drugs.com^®^ with 3 risk levels: major, moderate, and minor. DDIs classified as major or moderate were considered as clinically relevant, leading to PNI in collaboration with the prescriber to decide between a switch to an alternative treatment and continuation with personalized monitoring.

Therapeutic information on the medication plan, AEs and DDIs was shared via secure messaging with the community pharmacist and physician.

### 4.2. Endpoints

The main endpoint was RDI at month 6 after OAD initiation, i.e., the percentage of dose intensity received by the patient during the first 6 months of treatment compared to the standard dose intensity established by the summary of product characteristics (SPCs). We hypothesized that the dose received by the patient is the dose prescribed by the hematologist adjusted for time of withdrawal due to events such as surgery or AEs.

Secondary endpoints were RDI at months 1, 3, 9 and 12, corrected RDI (RDIc), PFS, OS, early treatment termination (ETT), AEs, adherence and DDIs. RDIc was defined as prescribed RDI corrected for modifications according to the SPC. ETT was defined as treatment terminated earlier than planned by the hematologist at inclusion (<6 months).

Medication adherence was assessed by medication possession ratio (MPR) and a 6-question self-administered “yes/no” questionnaire at each consultation, leading to 3 levels of compliance: maximum, intermediate and insufficient/non-compliance. MPR is the percentage of supply days received divided by the dispensing period. The questionnaire consisted of a series of 6 “yes/no” questions, self-administered at each consultation, scheduled for months 1, 3, 6, 9 and 12 according to the duration of the program. Three levels of compliance were set: maximum (only “no” answers), medium or intermediate (4 or 5 “no”) and insufficient or noncompliance (3 or less “no”).

AEs were detected during interviews and graded 1 to 5 according to the CTCAE version 4.0 [62].

### 4.3. Statistical Analysis

The population was described using numbers and percentages for categorical variables, and medians and inter-quartile ranges for continuous variables. OS and PFS were estimated on Kaplan–Meier plots.

To mitigate the impact of the immortal-time bias in the measurement of RDI, a landmark approach was employed to assess the evolution of RDI over time, whereby subjects who failed prior to a given landmark were excluded from the analysis. We considered landmarks at 30 days, 90 days, 180 days and 1 year. Because RDI was not normally distributed, the Friedman test was used to compare RDI at 30 days, 90 days and 180 days for patients at the landmark of 180 days.

Associations between patient characteristics and ETT were evaluated via univariate and multivariate logistic regression with Firth’s correction to take account of the small sample sizes and low prevalence [63]. A backward procedure, based on a penalized likelihood ratio test, was used for variable selection to detect the characteristics most predictive of ETT. The variables of interest comprised age, gender, living alone or not, CCI, polypharmacy (>5 medications), CAM use, pathology and comorbidities including hypertension and cardiovascular disease. A linear effect was considered for age and CCI. All tests were 2-sided, with *p* < 0.05 considered significant. Analyses were performed on R 4.0.3 software with the logistf package for logistic regression with Firth’s correction.

## 5. Conclusions

Patient education and multidisciplinary follow-up should be the gold standard for patients receiving OADs to ensure drug safety and risk management in clinical practice. RDI appears to be a reliable clinical quality indicator for treatment exposure, and can be used to assess the follow-up, as well as offering a predictive factor for early treatment termination, similar to age, gender, hematological malignancy, and Charlson Comorbidity Index. RDI provides real-life data on efficacy, dose reduction and treatment interruption or cessation; it is thus of interest for clinicians and health decision-makers. This model could be further extended to other chronic diseases requiring long-term oral medication. Although this study is monocentric and focuses on patients with hematological malignancies, Oncoral follow-up is being developed in this center for patients with all types of cancer (hematological malignancies and solid tumors), and is already being used as a model in a French national experiment involving 41 centers, for which the resources in terms of pharmacists and nurses are still being financed, which remains a necessary condition for the geographical extension of this type of program [64]. A medico-economic study is currently being carried out to evaluate the follow-up [61]. In addition, further research is needed to offer this assessment to other centers, both as part of routine care and as part of clinical trials, in order to harmonize practice and move towards a standard of care. Patients enrolled in clinical trials were not eligible for Oncoral follow-up for regulatory reasons, and a method for the multidisciplinary follow-up of clinical trial patients is being developed [65].

## Figures and Tables

**Table 1:** Patients’ characteristics at ibrutinib initiation according to diseases. Data are median (IQR) or n (%).

Pathology	Chronic Lymphocytic Leukemia (n = 49, 59%)	Mantel Cell Lymphoma (n = 18, 22%)	Waldenström (n = 16, 19%)	Total (n = 83)
Patient number evolution				
Month 1	49	17	16	82
Month 3	45	15	16	76
Month 6	42	10	12	64
Age, years				
Median (IQR)	71.07 (63.8–80.0)	78.00 (68.8–81.1)	77.53 (70.6–79.8)	74.38
Sex				
Female	17 (34.7%)	2 (11.1%)	7 (43.8%)	26 (31.3%)
Male	32 (65.3%)	16 (88.9%)	9 (56.2%)	57 (68.7%)
Charlson				
Median (IQR)	3.00 (2.00–4.00)	4.50 (4.00–5.00)	3.50 (3.00–4.25)	4.00
Hypertension	21 (42.9%)	5 (27.8%)	4 (25.0%)	30 (36.1%)
Cardio-vascular disease (CVD)	9 (18.4%)	4 (22.2%)	7 (43.8%)	20 (24.1%)
Treatment line				
1	6 (12.2%)	0 (0.0%)	1 (6.2%)	7 (8.4%)
2	26 (53.1%)	11 (61.1%)	7 (43.8%)	44 (53.0%)
3	11 (22.4%)	5 (27.8%)	1 (6.2%)	17 (20.5%)
4 and more	6 (12.2%)	2 (11.1%)	7 (43.8%)	15 (18.1%)

**Table 2:** Effects of concomitant drugs or CAM on ibrutinib in DDIs leading to PNIs.

Effect of Concomitant Drug and CAM	Drug and CAM	Interaction’s Classification According to Drugs^®^
	Apixaban	Major
	Rivaroxaban	Major
Increased risk of bleeding	Clomipramine	Moderate
	Diclofenac (topic et ophtalmique)	Moderate
	Duloxetine	Moderate
	Escitalopram	Moderate
	Fluoxetine	Moderate
	Paroxetine	Moderate
	Sertraline	Moderate
	Venlafaxine	Moderate
	Vitamine E	/
Enzyme inducer	Carbamazepine Phenobarbital	Major
		Major
	Diltiazem	Major
	Verapamil	Major
	Amiodarone	Moderate
Enzyme inhibitor	Turmeric	/
	Grapefruit	/
	Linseed oil	/
	Aloe vera	/
	Ginger	/
Modification of metabolism by ibrutinib	Loperamide	Moderate
	Silodosine	Moderate
	Sitagliptine	Moderate
Antioxidant, reduction in absorption or other mechanism	Omega 3/6	/
	Propolis	/
	Psyllium	/
	Spirulina	/

**Table 3:** Patient characteristics according to RDI at day 180.

180 Day RDI	RDI < 90%	RDI ≥ 90%	Total
	N = 13	N = 51	N = 64
age			
Mean (SD)	73.43 (8.95)	70.59 (10.13)	71.17 (9.90)
Median	75.50	70.44	71.03
Q1–Q3	68.35–80.01	64.06–78.10	65.20–79.68
Min-Max	50.18–84.08	50.10–88.77	50.10–88.77
N	13	51	64
Sex			
F	7 (53.8%)	17 (33.3%)	24 (37.5%)
M	6 (46.2%)	34 (66.7%)	40 (62.5%)
Charlson			
Mean (SD)	3.92 (1.66)	3.45 (1.91)	3.55 (1.86)
Median	4.00	3.00	3.50
Q1–Q3	3.00–4.00	2.00– 4.50	2.00– 4.25
Min-Max	1.00–7.00	1.00–12.00	1.00–12.00
N	13	51	64
polypharmacy			
0–5	9 (69.2%)	38 (74.5%)	47 (73.4%)
5+	4 (30.8%)	13 (25.5%)	17 (26.6%)
CAM			
no	4 (30.8%)	12 (23.5%)	16 (25.0%)
yes	9 (69.2%)	39 (76.5%)	48 (75.0%)
Adverse events			
Grade 0, 1, 2	10 (76.9%)	36 (70.6%)	46 (71.9%)
Grade 3–4	3 (23.1%)	15 (29.4%)	18 (28.1%)
Live_alone			
0	8 (61.5%)	36 (70.6%)	44 (68.8%)
1	5 (38.5%)	14 (27.5%)	19 (29.7%)
NA	0 (0.0%)	1 (2.0%)	1 (1.6%)
Pathology			
CLL	7 (53.8%)	35 (68.6%)	42 (65.6%)
Mantel cell lymphoma	1 (7.7%)	9 (17.6%)	10 (15.6%)
Waldenström	5 (38.5%)	7 (13.7%)	12 (18.8%)

**Table 4:** Univariate and multivariate logistic regression with Firth’s correction upon early termination of ibrutinib (before 6 months).

				Univariate Logistic Regression			Multivariate Logistic Regression	
		Yes	No	OR [95% CI]	*p* Value		OR [95% CI]	*p* Value
Age	(continuous)	19	64	1.07 [1.01; 1.15]	0.02		1.10 [1.03; 1.19]	>0.01
Sex	Female	2	24	1 (reference)			1 (reference)	
	Male	17	40	4.23 [1.19; 22.59]	0.02		6.44 [1.65; 37.21]	0.01
Charlson	(discrete)	19	64	1.27 [1.01; 1.65]	0.04			
Polypharmacy	0–5	12	47	1 (reference)				
	5+	7	17	1.63 [0.55; 4.65]	0.37			
CAM use	No	5	16	1 (reference)				
	Yes	14	48	0.9 [0.30; 2.97]	0.85			
Live alone	No	14	44	1 (reference)				
	Yes	5	19	0.87 [0.26; 2.55]	0.80			
Pathology	CLL	7	42	1 (reference)				
	MCL	8	10	4.59 [1.4; 15.63]	0.01			
	WM	4	12	2.04 [0.51; 7.6]	0.30			
Hypertension	No	14	39	1 (reference)				
	Yes	5	25	0.59 [0.18; 1.7]	0.33			
Cardio-vascular disease	No	11	52	1 (reference)				
	Yes	8	12	3.1 [1.04; 9.23]	0.04			

OR, odds ratio; CLL, Chronic Lymphocytic Leukemia; MCL, Mantel cell lymphoma; WM, Waldenström macroglobulinemia.

## Data Availability

The data that support the findings of this study are available on request from the corresponding author C.R. The data are not publicly available due to legal and privacy reasons.
